# A Warning Concerning Drainage Tubes

**Published:** 1902-05-17

**Authors:** 


### A WARNING CONCERNING DRAINAGE TUBES.

A CASE is reported by Dr. Owen Rhys[1] of a man, aged 60, who was admitted to the Cardiff Infirmary with an old empyema of the right side. His illness had commenced two and a-half years previously, and a month later a small piece of the fifth rib on the right side had been resected. According to the patient's account, while the dressings were being changed by a nurse at his cottage, he noticed that only the end of the drainage tube, with the safety pin attached, could be seen among the dressings. The missing portion of the tube could not be found. On two subsequent occasions the same thing happened, and when admitted to the infirmary he stated that he had had three pieces of red rubber drainage tube in his chest for two years. On operation, at first nothing but pus could be found, but ultimately the three pieces of tubing were discovered and brought away. Six weeks after the operation the patient was discharged in good health, and with the wounds entirely healed.

[1] Brit. Med. Jour., May 3.

